# Isolation, Characterization, and Bioactivity Evaluation of 3-((6-Methylpyrazin-2-yl)methyl)-1*H*-indole, a New Alkaloid from a Deep-Sea-Derived Actinomycete *Serinicoccus profundi* sp. nov

**DOI:** 10.3390/md11010033

**Published:** 2012-12-27

**Authors:** Xian-Wen Yang, Gai-Yun Zhang, Jian-Xi Ying, Bing Yang, Xue-Feng Zhou, Andre Steinmetz, Yong-Hong Liu, Ning Wang

**Affiliations:** 1 Luxembourg Public Research Center for Health (CRP-SANTE), 84 Val Fleuri, Luxembourg L-1526, Luxembourg; E-Mails: xianwen.yang@crp-sante.lu (X.-W.Y.); andre.steinmetz@crp-sante.lu (A.S.); 2 Key Laboratory of Marine Bio-resources Sustainable Utilization, South China Sea Institute of Oceanology, Chinese Academy of Sciences, 164 West Xingang Road, Guangzhou 510301, China; E-Mails: bingo525@163.com (B.Y.); xfzhou@scsio.ac.cn (X.-F.Z.); 3 Key Laboratory of Marine Biogenetic Resources, Third Institute of Oceanography, State Oceanic Administration, Xiamen 361005, China; E-Mails: zhgyun2008@yahoo.com.cn (G.-Y.Z.); yingjianxiah@126.com (J.-X.Y.)

**Keywords:** deep-sea sediment, actinomycete, *Serinicoccus profundi* sp. nov., alkaloid, antibacterial activity, cytotoxicity

## Abstract

One new alkaloid, 3-((6-methylpyrazin-2-yl)methyl)-1*H*-indole (**1**) was obtained from the deep-sea actinomycete *Serinicoccus profundi* sp. nov., along with five known compounds (**2**–**6**). Their structures were determined on the basis of detailed analysis of the 1D and 2D NMR as well as MS data. The new indole alkaloid displayed weak antimicrobial activity against *Staphylococcus aureus* ATCC 25923 with an MIC value of 96 μg/mL. It showed no cytotoxicity on a normal human liver cell line (BEL7402) and a human liver tumor cell line (HL-7702).

## 1. Introduction

In recent years, many novel genera and species of actinomycetes have been isolated from marine sediments [1,2,3,4]. Subsequently, more and more secondary metabolites have been discovered with novel structures and potent bioactivities [5,6,7,8,9]. This, in turn, attracted more and more attention from scientists discovering novel compounds from marine-derived actinomycetes, especially those from deep-sea sediments.

**Figure 1:**
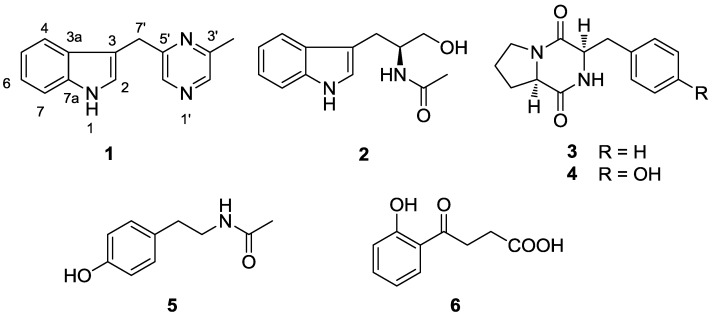
Compounds isolated from actinomycete *Serinicoccus profundi* sp. nov.

The genus *Serinicoccus* belongs to the family *Intrasporangiaceae*, suborder *Micrococcineae*. It was first described in 2004 [10]. Up to now only three species were found: *Serinicoccus profundi* sp. nov., *S. marinus* gen. nov., and *S. chungangensis* sp. nov. [10,11,12]. All of them were isolated from marine habitats. The type strain of *S. profundi* MCCC 1A05965^T^ was isolated from a deep-sea sediment of the Indian Ocean collected at a depth of 5368 m [12]. Herein, we describe the isolation and structural elucidation of a new indole alkaloid (**1**) from this actinomycete, together with five known compounds (**2**–**6**, Figure 1). Moreover, antimicrobial and cytotoxic activities are reported for compound **1**. This is the first report on the secondary metabolites from this genus.

## 2. Results and Discussion

### 2.1. Structure Elucidation

**Figure 2:**
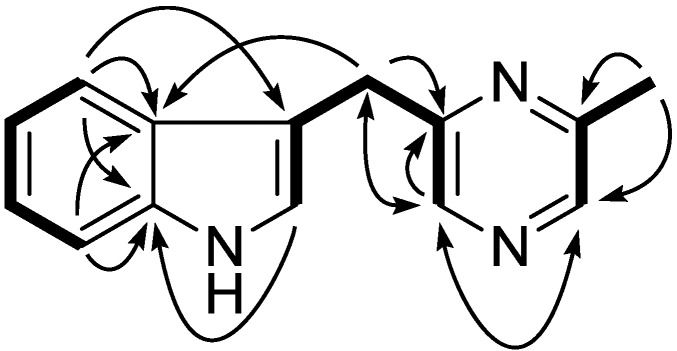
Key ^1^H–^1^H COSY (bold) and HMBC (arrow) correlations for compound **1**.

Compound **1** was isolated as a colorless liquid. Its molecular formula was deduced as C_14_H_13_N_3_ on the basis of its positive HR-ESI-MS pseudomolecular ion at *m/z* 224.1185 [M + H]^+^. The ^1^H NMR showed a typical 1,2-disubstituted benzoic moiety with signals at δ_H_ 7.10 (1H, dt, *J* = 8.1, 1.1 Hz), 7.20 (1H, dt, *J* = 8.1, 1.1 Hz), 7.37 (1H, d, *J* = 8.1 Hz), and 7.55 (1H, d, *J* = 8.1 Hz), in addition to eight other signals including three sp^2^ methine [7.08 (1H, m); 8.27 (2H, br s)], one methylene [4.28 (2H, s)], and one methyl group [2.56 (3H, s)]. The ^13^C and DEPT NMR spectra revealed the presence of seven sp^2^methine (111.2 d, 119.0 d, 119.6 d, 122.3 d, 122.6 d, 141.4 d, and 141.8 d), one sp^2^ methylene (32.1 t), and one methyl carbon (21.6 q). A further five sp^2^ quaternary carbons were observed in the ^13^C NMR spectrum at δ_C_ 112.9, 127.2, 136.4, 152.7, and 155.5. Altogether, the 1D NMR spectra gave fourteen carbons with one methyl, one methylene, seven methines, and five quaternary carbons (Table 1). In the ^1^H–^1^H COSY spectrum, three spin systems were found which constructed three fragments of C-4/C-5/C-6/C-7, C-2/C-3/C-7′/C-5′/C-6′, and C-2′/C-3′/C-8′. These three fragments could easily be connected according to the key HMBC correlations of H-4 to C-3/C-3a/C-7a, H-2 to C-7a, H-2′ to C-6′, and H-6′ to C-2′/C-5′/C-7′, which established the whole structure as shown in Figure 2. Further confirmation was found for HMBC correlations of H-8′ to C-3′/C-2′, H-7′ to C-5′/C-6′/C-2/C-3/C-3a, H-4 to C-3/C-3a/C-7a, and H-7 to C-3a/C-7a. On the basis of the above evidence, compound **1** was assigned as 3-((6-methylpyrazin-2-yl)methyl)-1*H*-indole.

**Table 1:** ^1^H and ^13^C NMR spectroscopic data for compound **1** in CDCl_3_.

No.	δ_C_	δ_H_
2	122.6 d	7.08 m
3	112.9 s	
3a	127.2 s	
4	119.0 d	7.55 (d, 8.0)
5	119.6 d	7.10 (dt, 7.6, 1.0)
6	122.3 d	7.20 (dt, 7.6, 1.1)
7	111.2 d	7.37 (d, 8.1)
7a	136.4 s	
2′	141.8 d	8.27 br s
3′	152.7 s	
5′	155.5 s	
6′	141.4 d	8.27 br s
7′	32.1 t	4.28 s
8′	21.6 q	2.56 s

By comparing NMR and MS data with reference data, five known compounds were identified as: (*S*)-α-acetylamino-β-(3-indole)propanol (**2**) [13], *cyclo*-(L-Pro-L-Phe) (**3**) [14], *cyclo*-(L-Pro-L-Tyr) (**4**) [15], *N*-(4-hydroxyphenethyl)acetamide (**5**), and 4-(2-hydroxyphenyl)-4-oxobutanoic acid (**6**).

### 2.2. Bioactive Tests

Compound **1** was tested for cytotoxic activity against a normal human liver cell line (HL-7702) and another human liver tumor cell line (BEL7402) at a concentration of 50 μg/mL. However, only weak activities were found with the inhibitory rate of 6.3% on HL-7702 and 1.7% on BEL7402. Moreover, compound **1** was further subjected to an antibacterial activity test against four different bacterial strains of *Staphylococcus aureus* ATCC 25923, *Bacillus thuringiensis*, *Escherichia coli* ATCC 35218, and *Bacillus subtilis* CMCC63501. It exhibited a weak activity on *Staphylococcus aureus* ATCC 25923 with an MIC value of 96 μg/mL, while no inhibitory activity was found against *Bacillus subtilis* CMCC63501, *Bacillus thuringiensis* ATCC 10792, and *Escherichia coli* ATCC 35218 with MIC values of around 200 μg/mL.

## 3. Experimental Section

### 3.1. General Experimental Procedures

NMR spectra were recorded on a Bruker Avance III 400 MHz NMR spectrometer with TMS as internal standard. ESIMS were measured on a Bruker amazon SL spectrometer, and HRESIMS were measured on a Bruker En Apex ultra 7.0T FT-MS spectrometer. UV spectroscopic data were obtained on a UV-2100 PC spectrophotometer. Materials for CC were silica gel (Huiyou Silical Gel Development Co. Ltd., Yantai, China), Sephadex LH-20 (Amersham Pharmacia Biotech AB, Uppsala, Sweden), and YMC-GEL ODS-A (YMC, USA). Preparative TLC was conducted with glass precoated silica gel GF254 (Yantai, China).

### 3.2. Microbial Source and Culture Conditions

The microorganism MCCC 1A05965^T^, identified as *Serinicoccus profundi* sp. nov., was isolated from a deep-sea sediment of the Indian Ocean, and was deposited in the Marine Culture Collection of China, China General Microbiological Culture Collection Center and German Collection of Microorganisms and Cell Cultures [12].

A single colony of MCCC 1A05965^T^ was inoculated into 50 mL of seed medium (soybean meal 0.3%, yeast extract 0.3%, trehalose 1%, proline 0.1%, beef extract 0.3%, glycerol 0.6%, K_2_HPO_4_ 0.05%, MgSO_4_·7H_2_O 0.05%, FeSO_4_·7H_2_O 0.05%, CaCO_3_ 0.2%, and sea salt 3% at pH 7.4 before sterilization) in 250 mL Erlenmeyer flasks, and was cultured on a rotary shaker at 200 rpm at 28 °C for 2 days. A total of 10 mL of seed cultures were transferred into 100 mL production medium (the same as the seed medium) in 500 mL Erlenmeyer flasks, and were cultured on a rotary shaker at 200 rpm at 28 °C for 6 days. 

### 3.3. Extraction and Isolation

After 6 days of cultivation, the broth was centrifuged to separate the mycelia cake from the fermentation liquid. The liquid (10 L) was extracted by ethyl acetate (EtOAc, 10 L) for three times to afford residue A after removal of the solvent. The mycelia cake was extracted three times with acetone (1 L). After concentration under reduced vacuum, the residue was re-extracted with EtOAc (1 L) to give residue B. Based on the results of HPLC and TLC analysis, residues A and B were combined as the crude extract.

The total crude extract (8 g) was subjected to column chromatography (CC) over silica gel using gradient CHCl_3_–MeOH (0%–100%), which afforded 5 fractions (Fr.1–Fr.5). Fraction Fr.1 was further purified by CC over silica gel eluting with petroleum ether (PE)-EtoAc (50:1). Final purification by CC over Sephadex LH-20 (CHCl_3_–MeOH, 1:1) gave **3** (28.5 mg). From fraction Fr.2, repeated CC over silica gel (CHCl_3_–MeOH, 50:1; PE-EtOAc, 10:1) and Sephadex LH-20 (CHCl_3_–MeOH, 1:1) provided **1** (4.8 mg). Similarly, **6** (5.8 mg) was obtained from fractions Fr.3 and Fr.4, respectively, after repeated CC over silica gel (CHCl_3_–MeOH, 15:1 for **6** as well as PE-EtOAc, 10:1) and Sephadex LH-20 (CHCl_3_–MeOH, 1:1). Fraction Fr.5 was first separated as three subfractions (Fr. 5.1–Fr. 5.3) by CC over sephadex LH-20. After CC over silica gel with CHCl_3_–MeOH (10:1) and further purification by CC on ODS with 45% MeOH, **2** (23.0 mg) was then obtained from subfraction Fr. 5.1. From subfraction Fr. 5.2, **4** (83.6 mg) was isolated after successive CC on silica gel eluted by PE-EtOAc (1:1) and CHCl_3_-MeOH (9:1). Compound **5** was purified from subfraction Fr. 5.3 after CC over silica gel (CHCl_3_-MeOH, 8:1), followed by final purification by preparative TLC using the same eluent system.

3-((6-Methylpyrazin-2-yl)methyl)-1*H*-indole (**1**): Colorless oil; UV (CHCl_3_) 237.4, 275.4 nm; for^1^H and ^13^C NMR data, see Table 1; ESIMS (positive) *m/z* 224.2 [M + H]^+^; HRESIMS (positive) [M + H]^+^ *m/z* 224.1185, calcd for C_14_H_14_N_3_, 224.1182.

### 3.4. Cytotoxic Assay

Compound **1** was tested for cytotoxic activity against a human liver tumor cell line (HL-7702) and a normal human liver cell line (BEL7402) as described previously [16]. Briefly, two cell lines were seeded into a 96-well plate and kept in a humidified atmosphere of 5% CO_2_ and 95% air at 37 °C. After 24 h, compound **1**was added and the incubation continued for another 48 h. An MTT solution was then added to evaluate the cell viability by measuring the optical density of the color produced by MTT dye reduction with a microplate reader at 570 nm.

### 3.5. Antibacterial Activity

MIC values for compound **1 **were determined against four bacterial strains of *Staphylococcus aureus* ATCC 25923, *Bacillus thuringiensis* ATCC 10792, *Escherichia coli* ATCC 35218, and *Bacillus subtilis* CMCC63501 as described previously [17]. 

## 4. Conclusions

This is the first report on chemical constituents form *Serinicoccus profundi* sp. nov., a rare actinomycete from a deep-sea sediment. Six components were isolated and purified including two indole alkaloids (**1**, **2**), two diketopiperazines (**3**, **4**), and two phenolics (**5**, **6**). Compound **1 **is a new secondary metabolite whose skeleton consists of an indole and a pyrazine. It was tested for cytotoxic and antibacterial activities. However, only a weak antibacterial activity was found on *Staphylococcus aureus* ATCC 25923 with an MIC value of 96 μg/mL.
